# Shared Genes and Molecular Mechanisms between Nonalcoholic Fatty Liver Disease and Hepatocellular Carcinoma Established by WGCNA Analysis

**DOI:** 10.1055/s-0043-1768957

**Published:** 2023-07-10

**Authors:** Juan He, Xin Zhang, Xi Chen, Zongyao Xu, Xiaoqi Chen, Jiangyan Xu

**Affiliations:** 1 Traditional Chinese Medicine (ZHONG JING) School, Henan University of Chinese Medicine, Zhengzhou, Henan, People's Republic of China; 2 First School of Clinical Medicine, Henan University of Chinese Medicine, Zhengzhou, Henan, People's Republic of China

**Keywords:** WGCNA, nonalcoholic fatty liver disease, hepatocellular carcinoma, genes, bioinformatics

## Abstract

**Background** Hepatocellular carcinoma (HCC) is one of the leading causes of death from cancer worldwide. The histopathological features, risk factors, and prognosis of HCC caused by nonalcoholic fatty liver disease (NAFLD) appear to be significantly different from those of HCC caused by other etiologies of liver disease.

**Objective** This article explores the shared gene and molecular mechanism between NAFLD and HCC through bioinformatics technologies such as weighted gene co-expression network analysis (WGCNA), so as to provide a reference for comprehensive understanding and treatment of HCC caused by NAFLD.

**Methods** NAFLD complementary deoxyribonucleic acid microarrays (GSE185051) from the Gene Expression Omnibus database and HCC ribonucleic acid (RNA)-sequencing data (RNA-seq data) from The Cancer Genome Atlas database were used to analyze the differentially expressed genes (DEGs) between NAFLD and HCC. Then, the clinical traits and DEGs in the two disease data sets were analyzed by WGCNA to obtain W-DEGs, and cross-W-DEGs were obtained by their intersection. We performed subsequent Gene Ontology (GO) and Kyoto Encyclopedia of Genes and Genome (KEGG) enrichment analyses of the cross-W-DEGs and established protein–protein interaction networks. Then, we identified the hub genes in them by Cytoscape and screened out the final candidate genes. Finally, we validated candidate genes by gene expression, survival, and immunohistochemical analyses.

**Results** The GO analysis of 79 cross-W-DEGs showed they were related mainly to RNA polymerase II (RNAP II) and its upstream transcription factors. KEGG analysis revealed that they were enriched predominantly in inflammation-related pathways (tumor necrosis factor and interleukin-17). Four candidate genes (JUNB, DUSP1, NR4A1, and FOSB) were finally screened out from the cross-W-DEGs.

**Conclusion** JUNB, DUSP1, NR4A1, and FOSB inhibit NAFLD and HCC development and progression. Thus, they can serve as potential useful biomarkers for predicting and treating NAFLD progression to HCC.

## Introduction

Hepatocellular carcinoma (HCC) is the most common pathological type of primary hepatic carcinoma 1 and one of the leading causes of death from cancer worldwide. 2 The incidence of HCC is closely related to factors such as hepatitis B or C virus infection, alcoholic liver injury, and obesity that lead to chronic liver disease and cirrhosis. Despite the existence of preventive measures, the overall incidence of HCC remains high. 3 Nonalcoholic fatty liver disease (NAFLD) is found to have affected approximately 25% of the global population and is one of the most common chronic liver diseases. 4 NAFLD includes two distinct pathological states, simple nonalcoholic fatty liver (NAFL) and nonalcoholic steatohepatitis (NASH), which have a different prognosis. The latter is the main cause of end-stage liver diseases such as liver fibrosis, liver cirrhosis, and liver cancer. 5 Existing clinical evidence and experimental data have confirmed that NAFLD is a high-risk factor for HCC, 6 7 but the research exploring the causative relationships between NAFLD and HCC is still in its early stage. To date, the histopathological features, risk factors, and prognosis of HCC caused by NAFLD have been found to be significantly different from those of HCC caused by other liver disease etiology, such as hepatitis B or hepatitis C virus infection. 8

Recent advances in high-throughput sequencing and bioinformatics analysis have enabled the identification of key target pathways at the molecular level, thereby facilitating the interpretation of the potential molecular mechanism for NAFLD evolution into HCC. Initial research in this direction has been initiated in the past few years. For instance, using gene microarrays, Cai et al identified seven differentially expressed genes (DEGs) in NAFLD and HCC, including *CDK1* , *HSP90AA1* , *MAD2L1* , and *PRKCD* . 9 Additionally, Ye et al discovered by Affymetrix microarray that *KIAA1462* was overexpressed in NASH-HCC tissues and it could interact with *LATS2* , a key molecule in the Hippo signaling pathway, to promote tumor development. 10 However, the DEGs of NAFLD and HCC identified in these studies have not been analyzed and discussed in association with the clinical characteristics of disease samples. There are slightly unclear in the discussion.

Weighted gene co-expression network analysis (WGCNA) is an emerging method applied to analyze the association between multiple sample genes and specific traits or phenotypes. In this study, we used WGCNA analysis to explore the key genes involved in the progression of NAFLD to HCC. We also validated the candidate genes by gene expression, survival, and immuno-histochemical analyses.

## Materials and Methods

### Data Acquisition, Preprocessing, and Standardization

The gene chip information of NAFLD was screened in the Gene Expression Omnibus (GEO) database ( https://www.ncbi.nlm.nih.gov/geo/ ), and the RNA-seq data of HCC was downloaded from the The Cancer Genome Atlas (TCGA) database ( https://portal.gdc.cancer.gov /). We first set screening criteria to select a suitable NAFLD data set for subsequent differential gene and WGCNA analyses: (1) the samples in the data set were of *Homo sapiens* , which were to be analyzed by high-throughput sequencing for expression profiling; (2) NAFLD in the samples in the data set had to be confirmed by pathological diagnosis; (3) a normal liver tissue in the data set had to serve as a control; and (4) the number of samples in the data set had to be ≥ 20. The NAFLD data sets in the GEO database were preliminarily screened based on these retrieval criteria. As of July 20, 2022, we obtained four eligible NAFLD data sets (GSE185051, GSE130970, GSE126848, and GSE135251). Meanwhile, we selected one HCC data set (GSE148355) and one NAFLD-related HCC data set (GSE192959, whose control tissues were derived from data set GSE193080) to be used for subsequent hub gene expression verification. The screening conditions of these data sets were consistent with those applied for the NAFLD data set, except for disease diagnosis ( Table 1 ).

**Table 1:** Eligible relevant data sets within the GEO database

GSE no.	No. of samples	Title	PMID
GSE185051	N = 5 vs. D = 52	Hepatic transcriptome profiling of a multiethnic cohort of pediatric non-alcoholic fatty liver disease patients reveals novel genes and pathways associated with disease stages	35312185
GSE130970	N = 6 vs. D = 72	Gene expression predicts histological severity and reveals distinct molecular profiles of nonalcoholic fatty liver disease	31467298
GSE126848	N = 14 vs. D = 16 vs. O = 15	Hepatic transcriptome signatures in patients with varying degrees of nonalcoholic fatty liver disease compared with healthy normal-weight individuals	30653341
GSE135251	N = 10 vs. D = 206	Transcriptomic profiling across the spectrum of nonalcoholic fatty liver disease	33268509
GSE148355	N = 15 vs. D = 62	Preoperative immune landscape predisposes adverse outcomes in hepatocellular carcinoma patients with liver transplantation	33772139
GSE192959 and GSE193080	N = 59 vs. D = 48	Transcriptome profile of liver biopsy tissues from patients with nonalcoholic fatty liver disease	35731891

Abbreviations: GEO, Gene Expression Omnibus; PMID, PubMed Identifier.

Note: “N”: Normal, “D”: Disease, “O”: Obese; GSE192959 and GSE193080 are part of the Super Series GSE193084.

Since the gene expression matrix and the clinical information of the samples in the TCGA database have been corrected, the preprocessing and normalization of these data were considered only for the data set information obtained in the GEO database. We downloaded and organized the gene expression matrices and sample clinical information of seven data sets from the GEO database, combined with the specific circumstances of the gene matrix in the data and the completeness of the sample clinical information. Finally, we selected the data set GSE185051 as the operational object of this analysis. Before performing differential gene analysis, we eliminated blank and abnormal information within GSE185051 and normalized it.

### Identification and WGCNA Analysis of DEGs

The standardized NAFLD data set GSE185051 was subjected to differential gene analysis using the R software “limma” package. Then, differential gene analysis with the R software “edgeR” package was performed on the HCC gene expression matrix. As a result, we identified the DEGs of NAFLD and HCC. Next, the DEGs of NAFLD and HCC and the clinical traits of the disease were analyzed by WGCNA to obtain the gene module (W-DEGs) with the highest degree of association with the disease; the intersection was taken to obtain the cross-W-DEGs. The WGCNA analysis in this study was performed on the Sangerbox bioinformatics analysis platform ( http://vip.sangerbox.com/home.html ).

### Enrichment Analysis of Cross-W-DEGs

The OmicShare Tool ( https://www.omicshare.com/tools ) is an online data analysis Web site, which we used to perform gene ontology (GO) and Kyoto Encyclopedia of Genes and Genome (KEGG) enrichment analysis of the cross-W-DEGs identified in this study. It is also worth noting that some of the genes in the cross-W-DEGs had the opposite expression status in NAFLD and HCC, and thus gene expression annotation was not performed in this enrichment analysis.

### Protein–Protein Interaction Network Construction and Correlation Module Analysis

STRING ( https://cn.string-db.org/ ) is a database for searching known and predicted PPIs, which we used to construct the PPI of the cross-W-DEGs network. The minimum required interaction score was set to ≥ 0.4. Then, the PPI network of cross-W-DEGs was subsequently imported into Cytoscape software for visualization optimization. Meanwhile, the “MCODE” plugin of Cytoscape was employed to analyze the association module of cross-W-DEGs to identify the key subnetworks and genes in the PPI network.

### Identification of Hub and Candidate Genes

We calculated the core hub genes in the PPI network using different algorithms built into the “CytoHubba” plugin of Cytoscape. Next, we listed the hub genes jointly analyzed by several algorithms as candidate genes. The candidate in the key modules was analyzed with “MCODE.” Then, we selected the final candidate genes among them based on our results and literature search.

### Verification of Candidate Genes

The NAFLD data set GSE135251, the HCC dataset GSE148355, and the NAFLD-related HCC data sets GSE192959 and GSE193080 in the GEO database were used to verify the expression of the candidate genes. We extracted data on the expression of each of the candidate genes in each data set and statistically analyzed the expression of the candidate genes in normal and tumor tissues. The survival information of HCC patients in the TCGA database was utilized to perform Kaplan–Meier (KM) survival analysis validation of the candidate genes. Immunohistochemical confirmation of the candidate genes was performed in the Human Protein Atlas.

## Results

### Specific Content of NAFLD and HCC Gene Data

**Fig. 1:**
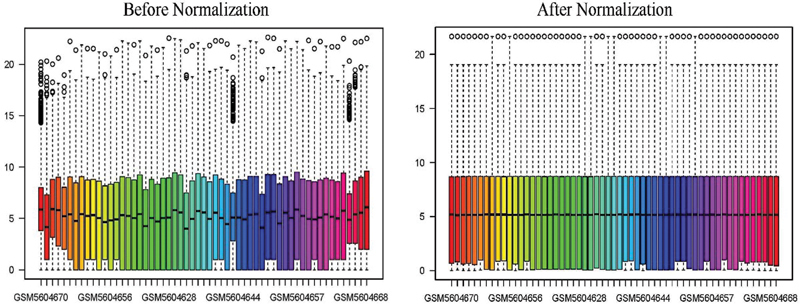
Boxplots of data set GSE185051 before and after normalization. The abscissa represents the 57 samples in the data set, and the ordinate represents the gene expression level in the sample (if the expression level > 30, log2).

The data set GSE185051 contained the information of 26,446 detected genes, along with the corresponding clinical information of 5 normal tissues and 52 NAFLD tissues. After eliminating the blank and abnormal information in the gene matrix, we used the “normalize between arrays” function of R software to normalize and remove the batch effect caused by other factors, so that the normal tissue and the diseased tissue could become comparable. The results before and after standardization are presented in Fig. 1 . From the TCGA database, we obtained the sequencing information of 59,427 genes and the corresponding clinical information of 50 normal and 374 tumor tissues.

### DEGs Identification and WGCNA Analysis Results of NAFLD and HCC

Using R software version 4.2.0 (the same below), the “limma” package, we set the filter conditions as the log fold change (logFC) > 1.5 and the false discovery rate < 0.01 for the differential gene analysis of NAFLD, finally obtaining 3,198 DEGs of NAFLD. The “edgeR” package was used for the differential gene analysis of HCC with screening conditions as specified above; finally, 5,860 DEGs of HCC were obtained. We sorted out the clinical information of the NAFLD and HCC samples, respectively. The clinical information of NAFLD patients included the patient's sample attributes (NAFLD or normal), gender, age, and liver fibrosis stage (0–3). The clinical information of HCC patients included the patient's sample attributes (HCC or normal), gender, body weight, TNM stage, Child–Pugh classification of liver function (A–C), and neoplasm histologic grade (G1–G4). Then, the differential gene matrix and clinical information of NAFLD and HCC were uploaded to the Sangerbox Web site for WGCNA analysis.

**Fig. 2:**
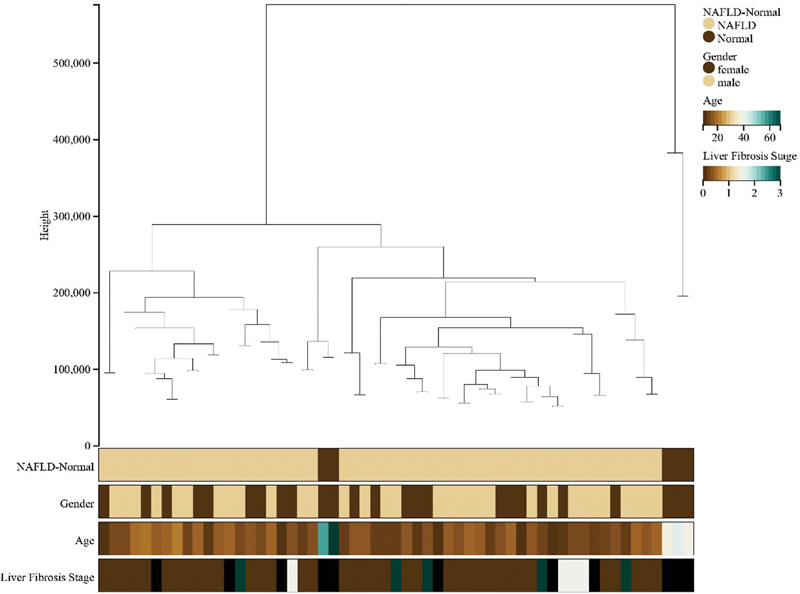
Sample clustering tree diagram for nonalcoholic fatty liver disease (NAFLD).

**Fig. 3:**
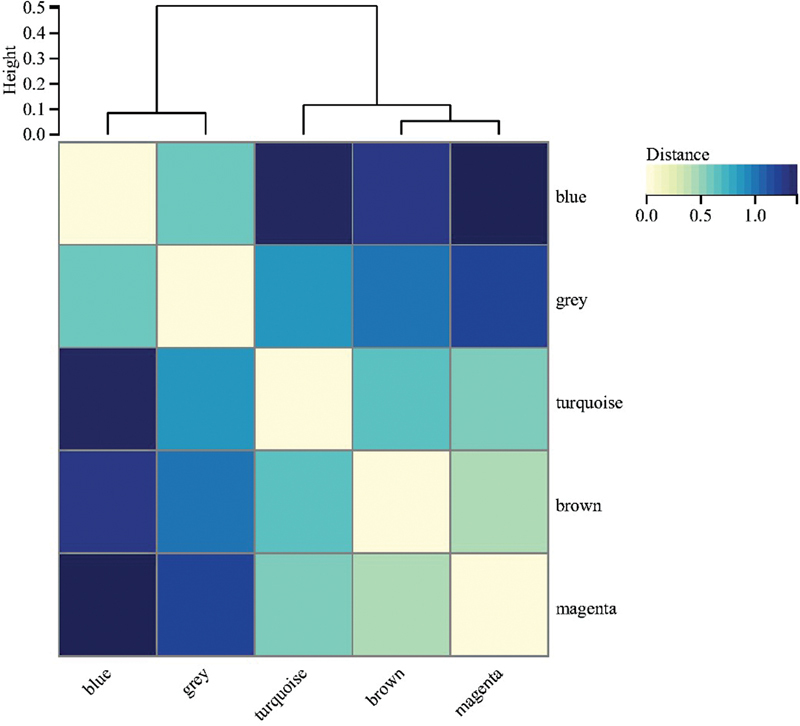
Cluster map of module eigenvectors for nonalcoholic fatty liver disease (NAFLD).

**Fig. 4:**
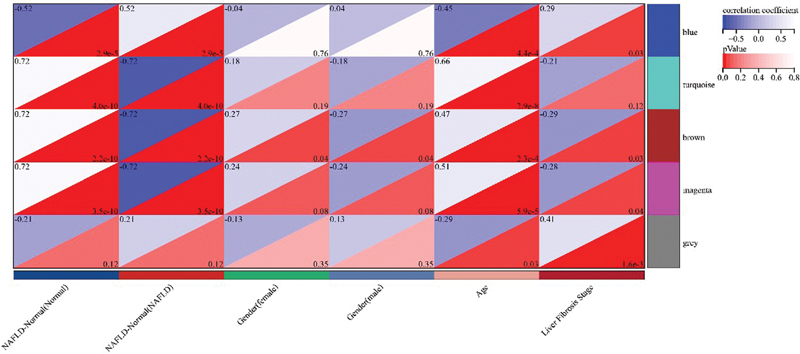
Heat map of module and phenotype correlations for nonalcoholic fatty liver disease (NAFLD).

**Fig. 5:**
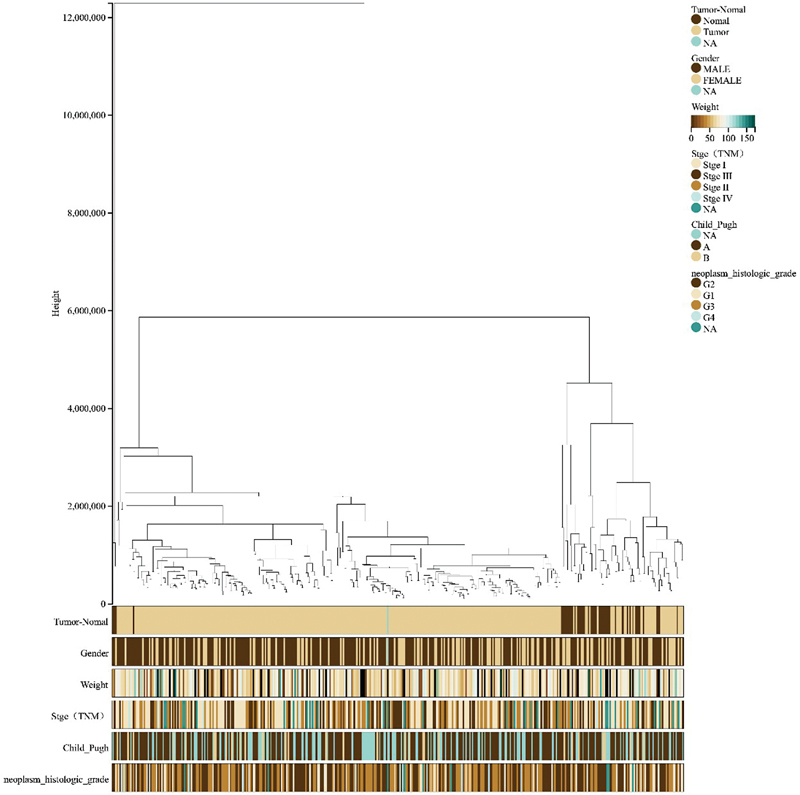
Sample clustering tree diagram of hepatocellular carcinoma (HCC). In the figure, above “0” is the sample clustering information of HCC or nonalcoholic fatty liver disease (NAFLD), different colors below “0” represent different phenotypes in different clinical information, and NA is a missing value.

**Fig. 6:**
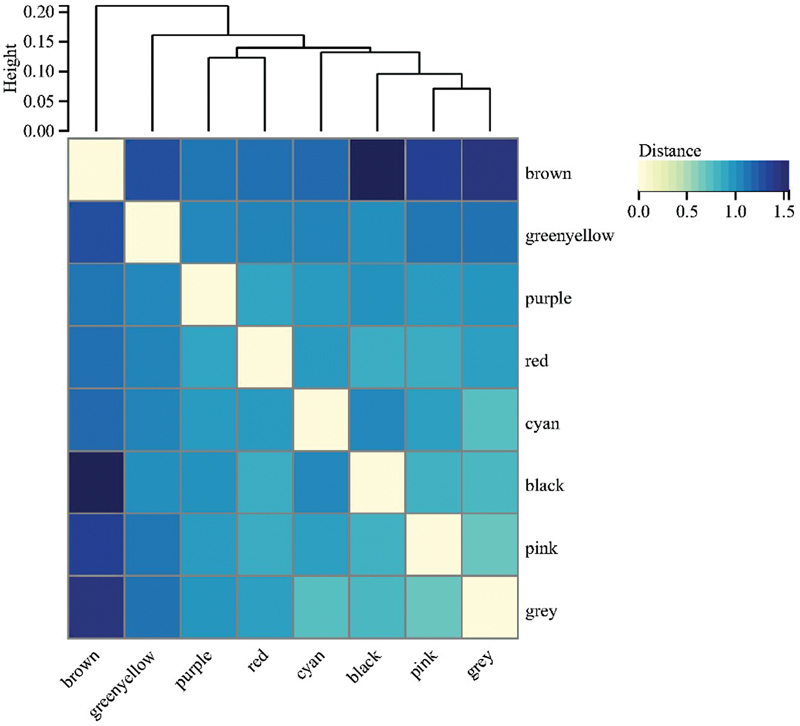
Cluster map of module eigenvectors for hepatocellular carcinoma (HCC). In the figure, above “0” is the gene module clustering information of HCC or nonalcoholic fatty liver disease (NAFLD), and the different colors of the intersection of the horizontal and vertical gene modules below “0” represent the different correlations between the modules.

**Fig. 7:**
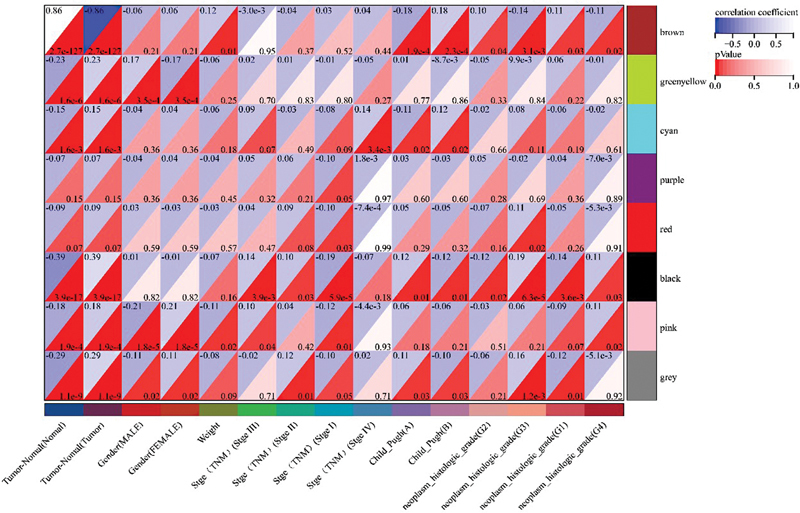
Heat map of module and phenotype correlations for nonalcoholic fatty liver disease (NAFLD). The upper part of the intersection of the clinical information on the horizontal axis and the gene module on the vertical axis in the figure is the correlation analysis coefficient of the two, and the lower part is the *p* -value of the correlation. The color of the intersection part changes based on the correlation coefficient and the *p* -value.

In the WGCNA analysis of NAFLD, we first clustered 57 samples in the NAFLD data set to obtain a sample clustering tree ( Fig. 2 ). Then, the DEGs of NAFLD were clustered based on the correlation parameter values ( *β* = 8) and the degree of dissimilarity between gene networks. The genes with similar expression profiles were classified into gene modules. The minimum value of the gene modules was set to 30, and the sensitivity value was set to 3. In addition, the modules with a distance of less than 0.25 were combined, obtaining five coexpression modules. The clustering diagram of the module feature vector is illustrated in Fig. 3 . Finally, we performed a correlation analysis between the genes of each module and the clinical information of NAFLD patients and drew a heat map of the correlation between modules and phenotypes, which can be seen in Fig. 4 . We selected a total number of 1,763 genes from the three modules of “brown (848),” “magenta (34),” and “turquoise (881)” with the highest degree of association with the disease as the W-DEGs of NAFLD. The WGCNA analysis steps of HCC were the same as described above. The sample clustering tree is presented in Fig. 5 . The soft threshold was set at 4. Since there were many differential genes in HCC, to prevent any influence on the obtained results, we increased the merging conditions of the gene module as follows: the minimum value of the gene modules was set to 80, the sensitivity to 3, and the module merging threshold to 0.75. The module eigenvector cluster map is depicted in Fig. 6 . The heat map of the correlation between the modules and the HCC phenotype is visible in Fig. 7 . Finally, we selected 1,077 genes in the “brown” module with the highest degree of disease association as the W-DEGs of HCC. Subsequently, 79 cross-W-DEGs were obtained by intersecting the W-DEGs of NAFLD and HCC.

### KEGG and GO Enrichment Analysis Results of Cross-W-DEGs

**Fig. 8:**
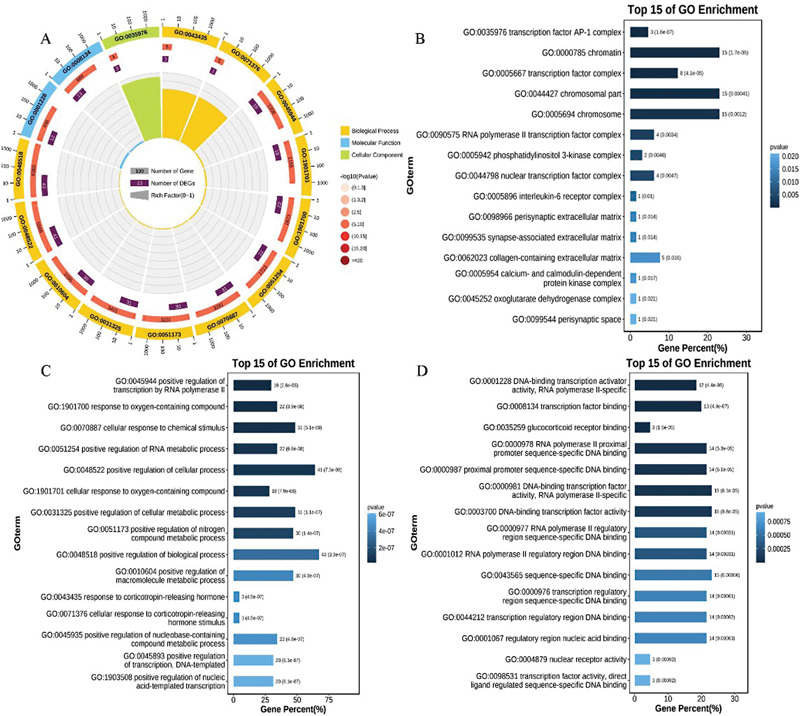
Gene Ontology (GO) enrichment analysis of the cross-W-differentially expressed genes (DEGs).

**Fig. 9:**
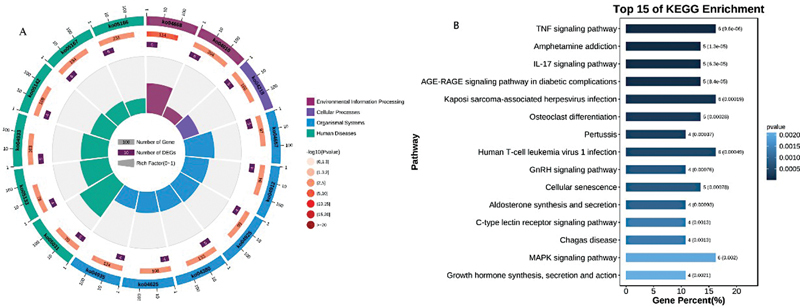
Kyoto Encyclopedia of Genes and Genome (KEGG) enrichment analysis of the cross-W-differentially expressed genes (DEGs).

The gene names of the cross-W-DEGs were converted into Ensembl IDs and uploaded to the OmicShare platform. Since some cross-W-DEGs showed opposite expression trends for the two diseases, we ignored the logFC value of the cross-W-DEGs, and finally chose the *p* -value for the plot; we visualized the top 15 GO or KEGG entries. GO enrichment analysis showed that cross-W-DEGs aggregated mainly in the biological process in three GO domains: biological process, molecular function, and cellular component. The top three main enriched functions are “positive regulation of transcription by RNA polymerase II (RNAP II),” “response to oxygen-containing compound,” and “DNA-binding transcription activator activity, RNAP II-specific,” as can be observed in Fig. 8 . KEGG enrichment analysis results showed that the cross-W-DEGs were clustered predominantly in organic systems and human diseases in the four categories of environmental information processing, cellular processes, organic systems, and human diseases. The top three main pathways enriched were the “tumor necrosis factor (TNF) signaling pathway,” “amphetamine addiction,” and “interleukin (IL)-17 signaling pathway” ( Fig. 9 ).

### PPI Network Construction and Correlation Module Analysis Results of the Cross-W-DEGs

**Fig. 10:**
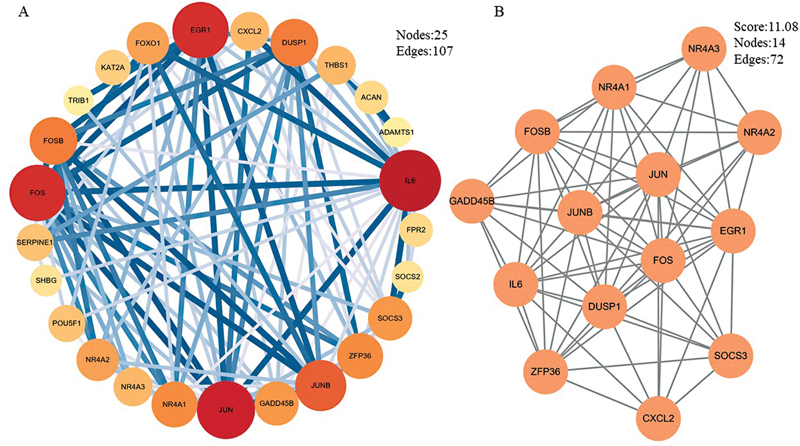
Diagram of the protein–protein interaction (PPI) network and associated modules of the cross-W-differentially expressed genes (DEGs). ( **A** ) The PPI optimization network of cross-W-DEGs. The size, color, and transparency of the module change according to the degree value, and the thickness and transparency of the line change according to the combined score value. ( **B** ) The key subnetworks in the cross-W-DEGs calculated by the MCODE module, the module scores, and the number of subnetwork nodes and connections are displayed in the figure.

On the STRING Web site, we constructed a PPI network of cross-W-DEGs including 70 points and 121 edges, and then imported the gene interaction results into Cytoscape software and calculated the degree value of the relationship between genes according to the degree value optimizing the display of the PPI network. The MCODE plugin in Cytoscape was used to analyze the key subnetworks and genes in the PPI network. The analysis criteria were set as follows: K-core = 2, degree cutoff = 2, max depth = 100, and node score cutoff value = 0.2. The visualization results and subnetworks of the PPI network are illustrated in Fig. 10 .

### Identification and Verification Results of Candidate Genes

**Fig. 11:**
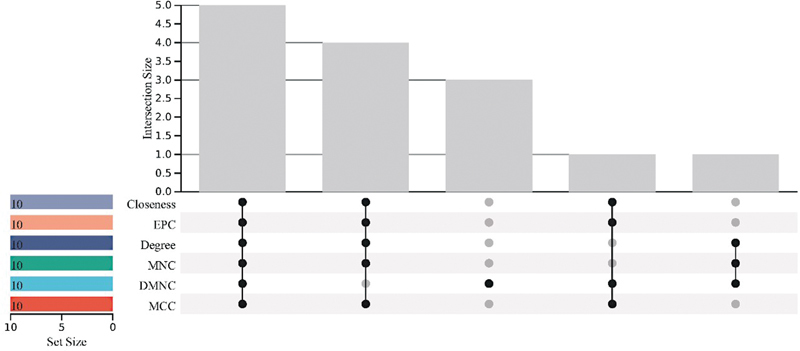
UpSet Venn diagram of the intersection genes of the six CytoHubba algorithms. In the figure, below “0” are six different algorithms of the CytoHubba plugin, and above “0” represents the number of intersecting genes identified by various algorithms.

The CytoHubba plugin in Cytoscape software was employed to analyze the interaction results of cross-W-DEGs to identify hub genes. We used its built-in six algorithms of MCC, DMNC, MNC, Degree, EPC, and Closeness to calculate the top 10 hub genes of cross-W-DEGs, respectively, took the intersection of them, and finally obtained five candidate genes ( Fig. 11 ). Then, based on our results and those obtained from literature research, we finally established and further used as candidate genes “JunB proto-oncogene, AP-1 transcription factor subunit (JUNB),” “dual specificity phosphatase 1 (DUSP1),” “FosB proto-oncogene, AP-1 transcription factor subunit (FOSB),” and “nuclear receptor subfamily 4 group A member 1 (NR4A1).” These four candidate genes also appeared in the key subnetwork obtained by MCODE.

**Fig. 12:**
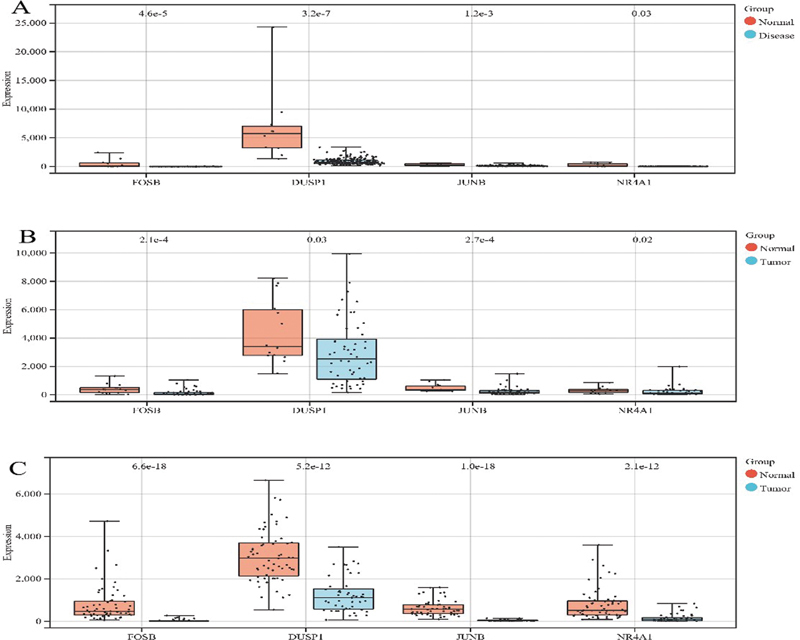
Boxplot for the expression level verification of the candidate genes. ( **A** ) The expression box plot of the candidate genes in the nonalcoholic fatty liver disease (NAFLD) data set GSE135251. ( **B** ) The expression box plot of the candidate genes in the hepatocellular carcinoma (HCC) data set GSE148355. ( **C** ) The expression box plot of the candidate genes in the NAFLD-related HCC data sets GSE192959 and GSE193080. The upper and lower lines of the boxplot represent the maximum and minimum expression values of the gene; the middle line indicates the median expression value of the gene; and the dots represent the samples.

**Fig. 13:**
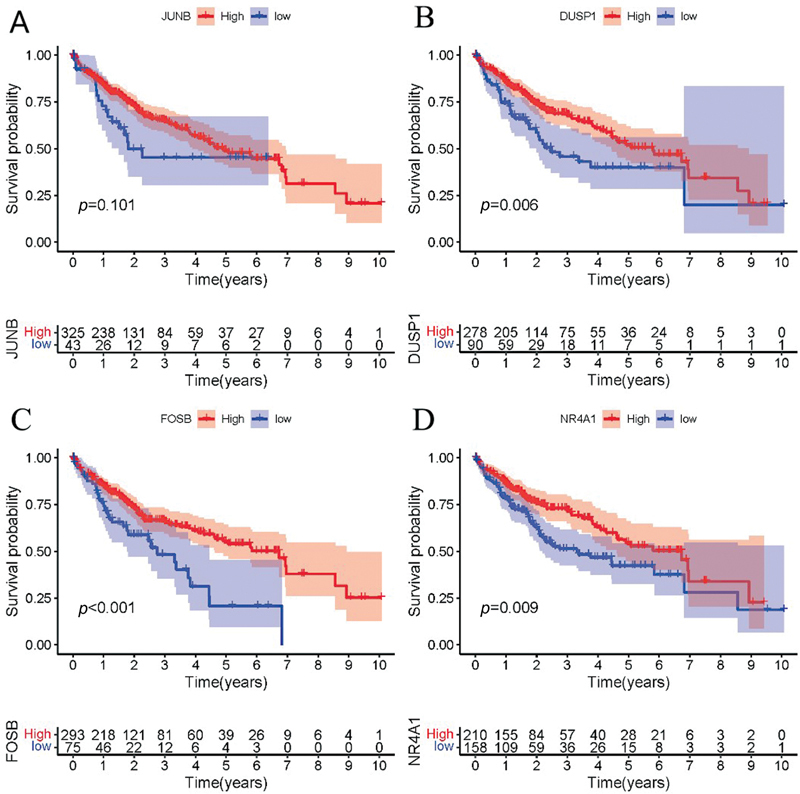
Kaplan–Meier (KM) survival analysis plot of the candidate genes. The lower coordinate axis in the KM analysis graph represents the number of samples corresponding to different risks and survival times.

**Fig. 14:**
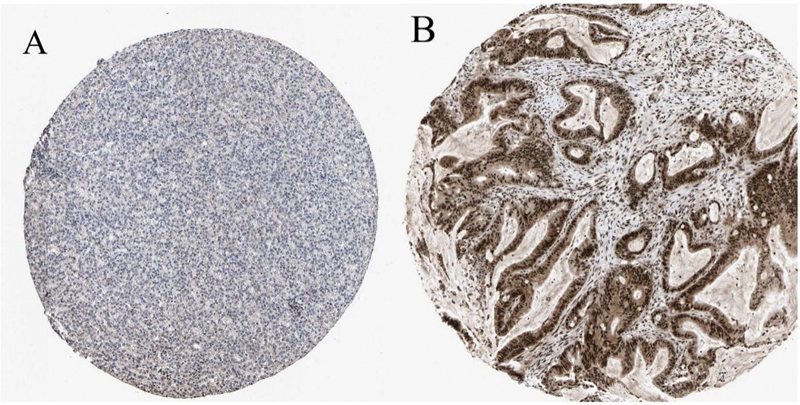
Immunohistochemistry of JUNB based on the Human Protein Atlas. ( **A** ) Protein levels of JUNB in hepatocellular carcinoma tissue (staining: low; intensity: weak; quantity: > 75%). ( **B** ) Protein levels of JUNB in normal tissue (staining: high; intensity: strong; quantity: > 75%).

We statistically analyzed the expression levels and grouping information of the four candidate genes in the NAFLD data set GSE135251, the HCC data set GSE148355, and the NAFLD-related HCC data sets GSE192959 and GSE193080. Parametric tests were performed if the data conformed to the normal distribution ( *t-* test); a nonparametric test (rank-sum test) was conducted for those that did not conform to the normal distribution. The results are presented in Fig. 12 . Significant differences ( *p* < 0.05) were found among the expression levels of JUNB, DUSP1, FOSB, and NR4A1 in the different groups of each data set. Then, we extracted the expression levels of the four candidate genes in each HCC sample and the overall survival corresponding to each sample, and arranged them into a matrix. Further, we performed a KM survival analysis of each gene. It is worth noting that for higher accuracy of the analysis results, we excluded the expression information of the normal samples; the KM analysis results are depicted in Fig. 13 . We established that in addition to JUNB, the survival rate corresponding to high and low expression of DUSP1, FOSB, and NR4A1 was significantly different ( *p* < 0.05). Therefore, the disease patients with high expression of DUSP1, FOSB, and NR4A1 may have a better survival prognosis. Finally, we verified JUNB in the Human Protein Atlas, the results of which showed that JUNB protein expression was significantly lower in the cancer tissues than in the adjacent normal tissues ( Fig. 14 ).

In Figs. 8 and 9A , from the outside to the inside, the first circle shows the specific GO or KEGG entries for the enrichment analysis; different colors represent different categories; the second circle represents the number of background genes and the enriched *p* -value; the third circle indicates the total number of foreground genes; the fourth circle shows the rich factor value (the number of foreground genes in the category divided by the number of background genes) of each category, where each small grid of the background auxiliary line is equal to 0.1.

Fig. 8B–D depicts bar graphs of the top 15 enriched entries of cross-W-DEGs in the three GO domains of biological process, cellular component, and molecular function in the GO enrichment analysis.

Fig. 9B depicts bar graph of the top 15 enriched entries of cross-W-DEGs in the KEGG enrichment analysis. The left side of the bar graph represents the specific description of the enriched function or pathway. The color and length of the graph to the right of the bar graph vary according to the number of gene enrichments and the *p* -value.

## Discussion

The incidence of NAFLD is increasing rapidly worldwide, and the number of people with NAFLD globally is expected to have increased by 56% by 2030. 11 12 Although the incidence of NAFLD-related HCC is lower than that of HCC caused by other chronic liver diseases such as hepatitis B and hepatitis C, we have to consider the seriousness of this problem due to the large population with this disease worldwide. 13 Currently, our understanding of HCC caused by NAFLD is still in its infancy. 14 Notably, no effective early diagnosis can currently be ensured during the process of NAFLD progression to HCC. Furthermore, the existing methods for the treatment of patients with NAFLD progression to HCC still do not provide a significant survival benefit. Therefore, further research on the molecular mechanism of NAFLD progression to HCC is critically needed, along with the exploration of new biomarkers that can provide a reference and the basis for the diagnosis, treatment, and prognosis of NAFLD-related HCC. To address this knowledge gap, we established a differential gene expression network between NAFLD and HCC based on WGCNA analysis results. Through the enrichment analysis of this network and the screening of key genes, the key pathway targets for the progression of NAFLD to HCC were obtained, and then the biological mechanism of its progression was explained at least in part.

In this study, we obtained 79 cross-W-DEGs associated with NAFLD and HCC disease via WGCNA analysis. Subsequent GO enrichment analysis revealed that cross-W-DEGs were related mainly to the functions of “positive regulation of transcription by RNAP II,” “response to oxygen-containing compound,” and “DNA-binding transcription activator activity, RNAP II-specific.” It is not difficult to see from the data that RNAP II and its upstream transcription factors play an important role in the progression of NAFLD to HCC. RNAP II is an enzyme in eukaryotic cells that catalyzes deoxyribonucleic acid transcription to synthesize messenger RNA (mRNA) and precursors of most heterogeneous nuclear RNAs and microRNAs. A recent study reported that “RNAP II subunit 3 (RPB3)” can promote the proliferation of HCC through targeted regulation of “vesicular, overexpressed in cancer, prosurvival protein 1 (VOPP1).” 15 In addition, in the present study, we found that RNAP II may be regulated by cyclin-dependent kinase (CDK) and cyclins to affect the occurrence and development of HCC. In a previous investigation, Yang et al established that cyclin L2 participated in pre-mRNA splicing through phosphorylation of the C-terminal domain of RNAPII and induced apoptosis in human hepatoma cells. Additionally, Zhong et al found that THZ1, an inhibitor of CDK7, induced apoptosis and inhibited the proliferation of liver cancer cells by reducing RNAPII-CTD phosphorylation, inducing p53 expression, and inhibiting anti-apoptotic gene expression. 16 Therefore, RNAPII and cell cycle-related factors may play an important unknown role in the progression of NAFLD to HCC, which needs to be confirmed. In addition, KEGG analysis of cross-W-DEGs showed that they were clustered mainly with the “TNF signaling pathway,” “amphetamine addiction,” and “IL-17 signaling pathway.” Hence, these genes are potentially linked to inflammation-related pathways, and it is well known that both NAFLD and HCC are diseases closely related to inflammation. In NAFLD, the activation of quiescent hepatic stellate cells and their differentiation into myofibroblasts are key events in the progression of NAFL to NASH. This differentiation process depends on the inflammatory activity of hepatic immune cells (mainly Kupffer cells). 17 18 HCC is a typical inflammatory cancer. The chronic inflammatory state of HCC is not only one of the important components of its complex tumor microenvironment, but it also affects the biological behavior of its metastasis and malignant prognosis. 19 Both TNF-α and IL-17 signaling pathways are canonical inflammatory pathways, and the results of several previous studies have evidenced their close connection with HCC. 20 21 Recent research has revealed the unique roles of these pathways in the progression of NAFLD to HCC. For example, Wu et al reported that the hepatocyte-specific deletion of the TNF family member TNF-α-induced protein 8-like 1 (TIPE1) exacerbated hepatic steatosis, inflammation, fibrosis, and systemic metabolic disturbances during the pathogenesis of NASH, which is an important pathological process in the progression of NAFLD to HCC. 22 Moreover, Serhal et al established that IL-17 was significantly increased in the serum of NASH patients as compared with that of healthy controls, and this increase was more pronounced in the serum of patients with NASH fibrosis. 23 These findings indicate that the inflammation-related TNF and IL-17 signaling pathways are not only a marker of the disease, but also a potential biological pathway for the progression of NAFLD to HCC.

We finally obtained four candidate genes, JUNB, DUSP1, NR4A1, and FOSB, by identifying the key genes of 79 cross-W-DEGs, which were verified at different levels. Interestingly, during the validation process, we found that JUNB, DUSP1, FOSB, and NR4A1 showed a basically consistent expression trend in NAFLD, HCC, and NAFLD-related HCC data sets; that is, the expression levels in normal control samples were much higher than those in diseased tissues. Meanwhile, the survival analysis of four candidate genes in TCGA data revealed that HCC patients with high expression of DUSP1, FOSB, and NR4A1 might have better survival prognoses. We searched the Human Protein Atlas for JUNB-associated negative survival analysis results and found that the protein expression of JUNB in normal samples was much higher than that in tumor samples. The aforementioned verification results not only confirm the accuracy of our results, but also verify that JUNB, DUSP1, FOSB, and NR4A1 play the role of tumor suppressor genes in the progression of NAFLD to HCC.

DUSP1, a member of the threonine-tyrosine dual-specificity phosphatase family, was first discovered in mouse cells; it was later found that the protein encoded by DUSP1 dephosphorylated the MAP kinases MAPK1/ERK2 in the MAPK signaling pathway. 24 The Ras/Raf/MAPK signaling pathway was activated in 50 to 100% of human HCC cases and was associated with poor prognosis. 25 The present study of DUSP1 in liver cancer initially revealed its specific mechanism of inhibiting the development of HCC.

Hao et al reported that DUSP1 increased the phosphorylation of p53 at the S15, S20, and S46 sites in HCC cells by inhibiting p38 MAPK phosphorylation, while the enhanced p53 activation induced the expression of the target genes p21 and p27, suggesting that DUSP1 and p53 may suppress HCC occurrence and development through the cooperation of active regulatory circuits. 26 Lopez-Yus et al reported that the expression of DUSP1 paralleled the degree of steatosis, and DUSP1 could be a key player in the progression of NAFLD. 27

JUNB, a member of the Fos/Jun family, is a key component of activator protein transcription factors and a major target element of mitotic activation and transmission pathways. 28 Previous studies have found that JUNB negatively regulated cell proliferation and Ras-mediated malignant transformation while also participating in proapoptotic pathways. 29 30 31 32 Guo et al found that the positive expression values of JUNB in tumor tissue and normal tissue were 20.00 and 76.32%, respectively. The dysfunctional p53 and low-expressed JUNB could synergistically downregulate the expression of the metastasis suppressor KAI1 in HCC, thereby affecting the survival prognosis of HCC patients. 33 Another study by Guo et al revealed that the fusion gene of wtp53 and JunB not only inhibited the growth of liver cancer cells and promoted tumor cell apoptosis, but also suppressed the invasive ability of tumor cells by upregulating the expression of KAI1. 34 FOSB is a member of the Fos gene family, which also includes FOS, FOSL1, and FOSL2. Many previous studies have reported the regulatory role of FOSB in the growth and metastasis of papillary thyroid cancer, 35 breast cancer, 36 nonsmall cell lung cancer, 37 prostate cancer, 38 and other tumors. Hu et al reported that interferon-α could reprogram glucose metabolism in HCC by acting on FosB, thereby unleashing T cell cytotoxic capacity and enhancing PD-1 blockade-induced immune responses. 39 JUNB and FOSB may act together on NAFLD. Activating protein-1 (AP-1) formed by the dimerization of proteins encoded by the Fos gene family and the JUN gene family may play an important role in the progression of NAFLD to HCC. Previous studies have confirmed that AP-1 can regulate gene expression in response to various external signals and is closely related to the regulation of various cellular processes, such as cell differentiation, proliferation, and apoptosis. 40 Hasenfuss et al reported that AP-1 was involved in acute stress response and fat metabolism in the liver under the form of a leucine zipper, which is an effective regulator of lipid metabolism and NAFLD development and plays an important role in obesity, liver lipid metabolism, and NAFLD. 41

NR4A1 is one of the members of the nuclear receptors subfamily 4 (NR4A) family. In recent years, accumulating evidence has shown that NR4A1 (Nur77) and other family members NR4A2 (Nurr1) and NR4A3 (Nor1) are critically involved in maintaining intracellular environmental stability and pathophysiology. 42 NR4A1 has been confirmed to be an oncogene in solid tumor in vivo studies, 43 44 45 but it appears to have the opposite prognostic profile in HCC. Notably, Guan et al found that NR4A1 was underexpressed in HCC, inhibiting cell proliferation, tumor growth, and tumor metastasis through the transcriptional activation of “lncRNA WAP four-disulfide core domain 21 pseudogene.” 46 Furthermore, He et al reported that NR4A1 acted as a key downstream mediator of Hippo-YAP signaling in HCC, participating in the proapoptotic function and antitumor effect induced by Hippo signaling in vitro and in vivo, promoting apoptosis and preventing tumorigenesis; the loss of NR4A1 promoted liver regeneration and tumorigenesis. 47 NR4A1 also plays an inhibitory role in NAFLD. Studies have found that NR4A1 can regulate the expression of adipogenesis-related genes, thereby suppressing adipocyte differentiation and adipogenesis. 48 In a high fat diet-fed mouse model, the knockout of NR4A1 aggravated insulin resistance and hepatic steatosis, whereas the overexpression of NR4A1 reduced hepatic triglyceride accumulation. 49

## Conclusion

The findings of the aforementioned studies suggest that JUNB, DUSP1, NR4A1, and FOSB are involved in inhibiting the growth and progression of both NAFLD and HCC. Therefore, they can be potentially useful biomarkers for the prediction and treatment of NAFLD-related HCC, which is consistent with our findings. However, experimental evidence is still needed to reveal their unique role in the progression of NAFLD to HCC or the treatment of NAFLD-related HCC.
